# Antifeedants Produced by Bacteria Associated with the Gut of the Pine Weevil *Hylobius abietis*

**DOI:** 10.1007/s00248-016-0915-5

**Published:** 2017-01-10

**Authors:** Karolin Axelsson, Vera Konstanzer, Gunaratna Kuttuva Rajarao, Olle Terenius, Lisa Seriot, Henrik Nordenhem, Göran Nordlander, Anna-Karin Borg-Karlson

**Affiliations:** 1 Royal Institute of Technology, School of Chemical Science and Engineering, Department of Chemistry, Ecological Chemistry Group, SE-100 44 Stockholm, Sweden; 2 Royal Institute of Technology, School of Biotechnology, Division of Microbiology, SE-106 91 Stockholm, Sweden; 3 Swedish University of Agricultural Sciences, Department of Ecology, P.O. Box 7044, SE-750 07 Uppsala, Sweden; 4 Institute of Technology, Division of Organic Chemistry, Nooruse 1, Tartu University, Tartu, Estonia

**Keywords:** Hylobius abietis, Rahnella, Brevundimonas, 2-Methoxyphenol, 2-Phenylethanol, Antifeedant

## Abstract

The pine weevil, *Hylobius abietis*, is a severe forest pest insect as it feeds on newly planted conifer seedlings. To identify and develop an antifeedant could be one step towards the protection of seedlings from feeding damage by the pine weevil. With the aim to trace the origin of the antifeedants previously found in feces of the pine weevil, we investigated the culturable bacteria associated with the gut and identified the volatiles they produced. Bacterial isolates were identified by 16S ribosomal RNA gene analysis. The volatile emissions of selected bacteria, cultivated on NB media or on the grated phloem of Scots pine twigs dispersed in water, were collected and analyzed by solid-phase microextraction gas chromatography-mass spectrometry. The bacterial isolates released a variety of compounds, among others 2-methoxyphenol, 2-phenylethanol, 3-methyl-1-butanol, 1-octen-3-ol, 3-octanone, dimethyl disulfide, and dimethyl trisulfide. A strong antifeedant effect was observed by 2-phenylethanol, which could thus be a good candidate for use to protect planted conifer seedlings against feeding damage caused by *H. abietis*.

## Introduction

The pine weevil, *Hylobius abietis* (L.) (Coleoptera: Curculionidae), is regarded as a severe pest of conifer reforestations in large parts of Europe and Asia [1]. The adult weevils feed on tender bark containing large amounts of terpenes and phenolics [2–4] of a wide range of conifer trees [5], with Scots pine (*Pinus sylvestris* (L.)) and Norway spruce (*Picea abies* (L.) H. Karst) as main hosts [6, 7]. Feeding weevils are especially destructive for newly planted conifer seedlings, which they frequently kill by girdling the stem [8].

Female weevils lay their eggs either in the soil near roots or in specific egg cavities gnawed into the root bark [9]. To construct the egg cavity, the female chews through the outer bark and continues into the phloem tissue along the xylem, about as far as can be reached by her snout. The egg is then deposited together with feces in the cavity, and the cavity is sealed with a plug made of frass (fragments nibbled from the outer bark) [10].

For *H. abietis*, the behavior to cover the eggs with feces and frass may protect the eggs from being eaten by other feeding pine weevils [9, 10]. The primary function of the frass and the feces might be that they emit antifeedants (substances that deter feeding by other pine weevils) and thus prevent conspecifics of either sex to feed close to existing egg cavities [9, 10].

It has been shown that chemical compounds in the feces from *H. abietis* inhibit the initiation of feeding by other individuals (both females and males) of the same species [9, 10]. Similarly, it has been shown that females of the granary weevil, *Sitophilus granarius* (L.), avoid feeding on grains with a sealed oviposition cavity, thereby avoiding infanticide [11]. The antifeedant activity in the solvent extract of *H. abietis* feces, and fractions thereof, was measured in a laboratory feeding bioassay by using twigs of Scots pine with both chemically treated and untreated bark surfaces. Based on these results, structure-activity studies have led to the identification of a large number of potent antifeedants [12–15].

Here, we hypothesize that the compounds responsible for this deterrent effect originate from microbes in the gut of *H. abietis*. The interest in understanding the role of bacteria associated with insects is increasing [16, 17]. The bacterial community in the insect gut has been shown to be a contributing factor for nutrition, protection from parasites and pathogens, immune responses, and communication [18]. Insects feeding on resources that are limited in nitrogen, e.g., wood and sap, may harbor nitrogen fixating bacteria that help the host by providing essential nutrition and vitamins [19, 20]. The influence of the bacterial community on the insect chemical communication and behavior [21] is now receiving increasing interest [22, 23]. For example, volatiles from fungi-infested feces have recently been shown to repel egg-laying flies [24], and volatiles as styrene, methylanisole, and methyl salicylate emitted from fungi isolated from pine weevil feces and frass have been shown to mask host volatiles and thereby alter the pine weevil orientation [25, 26]. Apart from these initial studies on fungi, the behavioral effect of volatiles emitted by pine weevil-associated microbes has not been investigated.

The present study focused on identifying possible antifeedant compounds produced by bacteria present in the gut of the pine weevil. Isolation of bacteria was made by culture-dependent methods, and the volatile compounds produced by these bacteria were identified by solid-phase microextraction gas chromatography-mass spectrometry (SPME-GC-MS). To elucidate the effects of the main bacterial volatiles on the pine weevils, antifeedant laboratory tests were made.

## Materials and Methods

### Pine Weevils

Both sexes of *H. abietis* were collected during spring migration at sawmills in southern Sweden, where they landed in large numbers. The weevils were stored at 10 °C and then placed individually for 2–7 days in Petri dishes with a moistened filter paper at 4 °C before they were used for isolation of bacteria. Weevils subsequently used in the antifeedant bioassay were stored in darkness at 10 °C and provided with fresh branches of Scots pine, *P. sylvestris*, as food. These storage conditions interrupted the reproductive development so that females did not begin oviposition until about a week after they had been transferred to the experimental conditions, i.e., a light regime of L18/D6 at 22 °C. This transfer was made about 10 days before the weevils were used in the bioassay.

### Microbial Isolation and Identification

Bacteria were isolated from the gut of the pine weevil. Five female weevils were surface-sterilized by dipping them individually in 70% ethanol followed by a rinse in phosphate buffer saline (PBS). The midgut and hindgut were homogenized aseptically in saline solution, diluted 1:10, 1:100, and 1:1000 times, spread (100 μl) on nutrient agar (NA) plates, and incubated for 24 h at room temperature, 30 °C and 37 °C. Single colonies growing on the NA plates were isolated, and pure cultures of the bacterial species were maintained on plates.

For identification of bacterial isolates, PCR of 16S was performed. One pure colony from each strain was boiled in 100 μl of water. The solution was vortexed, and then 1 μl was used for PCR in 25 μl reactions using Illustra PuRe Taq Ready-To-Go PCR Beads (GE Healthcare, Uppsala, Sweden). In each reaction, 0.4 μM each of forward and reverse 16S primers, 341f (5′-CCTACGGGNGGCWGCAG-3′) and 1401r (5′-CGGTGTGTACAAGACCC-3′), were used. The PCR program was as follows: 94 °C for 3 min, followed by 30 cycles of 94 °C for 30 s, 58 to 48 °C for 30 s (the temperature was decreased by 1 °C every cycle for 10 cycles and then held at 48 °C for 20 cycles), and 72 °C for 1 min, and followed by a final extension step at 72 °C for 20 min. Amplification products were sequenced at Macrogen (South Korea).

### Identification of Bacterial Headspace Volatiles on Different Media

Sixteen bacterial isolates were used for an initial screening of volatiles. The isolates were grown over night in 50 ml of nutrient broth (NB) in a 250-ml Erlenmeyer flask which was closed first with aluminum foil and then with parafilm. Three bacterial isolates (Ha1, Ha2, and Ha3) were selected for additional investigations based on their differences in volatile profiles. Media were chosen to represent different nutrient availabilities; (i) NB medium, (ii) 10 times diluted NB with 1% grated Scots pine phloem from twigs (natural nutrient), and (iii) 1% grated phloem (natural nutrient) in water only. A fourth medium, (iv) NB medium supplemented with ^13^C-6-ring, isotope-labeled L-phenylalanine (99%, Larodan), was used to confirm and characterize the ability of the bacterial isolates to utilize L-phenylalanine for the production of aromatic compounds. The incorporation of the carbon isotope in a compound produced by microbes was measured by the intensity of the M and M+6 fragments and by calculating the isotope ratio [(M+6)/M × 100], where *M* = molecular ion and M+6 is the molecular weight of the isotope containing molecular ion.

All the three isolates were grown overnight in NB medium (isolates Ha1 and Ha2 were incubated at 30°C and Ha3 was incubated at room temperature), sub-cultured in the respective nutrient media (mentioned above) with an optical density of 0.1 OD_600_ followed by further growth for 7 h, and analyzed the volatile production. The optical density of 7-h grown culture in NB was 0.8 OD_600_, and the cultures grown in 10 times diluted NB medium had an optical density of 0.3 OD_600_.

During the collection of volatiles, the cultures were kept at room temperature. A minimum of three replicates were analyzed for each isolate and medium. SPME StableFlex fibers coated with 65 μm, StableFlex, bonded polydimethylsiloxane/divinylbenzene (PDMS/DVB; Supelco Bellefonte, PA, USA) were used for collection of volatiles. The fibers were cleaned (sterilized) by heating in the GC injector for 5 min at a temperature of 220 °C and then promptly inserted through the aluminum foil in the top of the Erlenmeyer flask to collect the headspace volatiles for 3 h.

The volatiles were separated and identified using a GC-MS system consisting of a Varian 3400 GC connected to a Finnigan SSQ7000 MS instrument. A DB-wax capillary column was used (30 m, ID 0.25 mm, film thickness 0.25 μm). The temperature program of the GC oven was set to 40 °C for 1 min followed by an increase of 5 °C/min up to 225 °C, then isothermal for 12 min. Injector temperature was 220 °C and the split was closed for 30 s (splitless injection). Helium at 10 psi corresponding to 1 ml/min was used as the carrier gas. Mass spectra 30–400 m/z were obtained at 70 eV with an ion source at 150 °C. The mass spectra and retention times of the compounds were compared with those of authentic standards and mass spectra obtained from the NIST 2008 reference library and Mass finder version 3.

### Antifeedant Properties of Selected Compounds

To confirm the pine weevil antifeedant activity of the three major compounds identified in the emissions of isolated bacterial isolates, feeding tests were made by means of a two-choice laboratory bioassay, used in several previous studies [10, 12–15, 27]. 2-Phenylethanol and 2-methoxyphenol were tested in the concentrations 5, 25, and 50 mM, while for phenol only 50 mM was used.

For each test, 40 pine weevils were used (20 females + 20 males); they were starved for 24 h before the test period. Each weevil was placed in a Petri dish (142-mm diam.) and was provided with a Scots pine twig (prepared as described below) placed on a moistened filter paper. To restrict the area where the beetles are allowed to chew, 5-cm-long twigs of pine were first divided in halves and wrapped with aluminum foil. Then, two holes of 5 mm in diameter and 25 mm apart were punched using sharp-edged metal rings. When removing the aluminum foil inside the metal rings, a clear boundary was created to the rest of the bark. In one of these metal rings on each twig, 100 μl of 50, 25, or 5 mM methanol solution of the test compound was applied (test), and in the other ring, the same amount of pure solvent was applied (control). The following day, after the solvent had evaporated, the metal rings were removed and the bioassay started. After 6 and 24 h, the proportions of the treated bark area and control bark area, which had been consumed in each test twig, were recorded. The antifeedant effect measured for each compound was calculated by means of an antifeedant index (AFIa) based on the feeding area (a). AFIa = 100 × (*C* − *T*)/(*C* + *T*) , where *C* represents the mean area of control surfaces consumed and *T* the mean area of treated surfaces consumed. Positive values (up to a maximum of 100) reflect an antifeedant effect, whereas negative values (down to a minimum of −100) indicate a stimulant effect on feeding.

## Results

### Bacterial Isolation and Identification

Bacteria isolated from pine weevil guts were identified based on 16S ribosomal RNA (rRNA) gene sequencing analysis. Out of 16 isolates, 13 belonged to *Rahnella aquatilis* and the 3 other isolates were identified as *Brevundimonas nasdae*, *Kocuria kristinae*, and *Micrococcus terreus* (Table 1).

**Table 1:** Bacterial isolates

Isolate	Species name^a^	Percent identity	GenBank accession no.^b^
B1/Ha1	*R. aquatilis* ^c^	100	KU902037/KU902043^d^
B2/Ha2	*B. nasdae*	100	KU902038
B5	*R. aquatilis*	100	KU902039/KU902044^d^
B6	*K. kristinae*	100	KU902040
B11	*M. terreus*	100	KU902041
C3/Ha3	*R. aquatilis*	100	KU902042/KU902045^d^

^a^Best hit in BLASTn with a species name. GenBank accessed on 14 March 2016

^b^GenBank accession numbers apply to 16S rRNA sequences of the isolates unless specifically noted

^c^In total, 13 isolates of *R. aquatilis* were retrieved. The ones listed here are those referred to in the paper

^d^rpoB sequences showing 97% identity between isolates B1, B5, and C3

### Identification of Volatile Organic Compounds from Bacteria in Different Media

After an initial screening of all isolates for headspace volatiles, three isolates were chosen for further analysis based on both their large differences in volatile profiles when growing in NB medium and their emission of potential antifeedant aromatic compounds. Two of the isolates were identified as *R. aquatilis* (H1 and H3) and one as *B. nasdae* (H2). One isolate identified as *K. kristinae* released a variety of ketones with 2-tetradecanone as the main compound, but as no aromatic compounds such as 2-phenylethanol was found in the headspace, the isolate was not used for further analysis (Table 1).

**Fig. 1:**
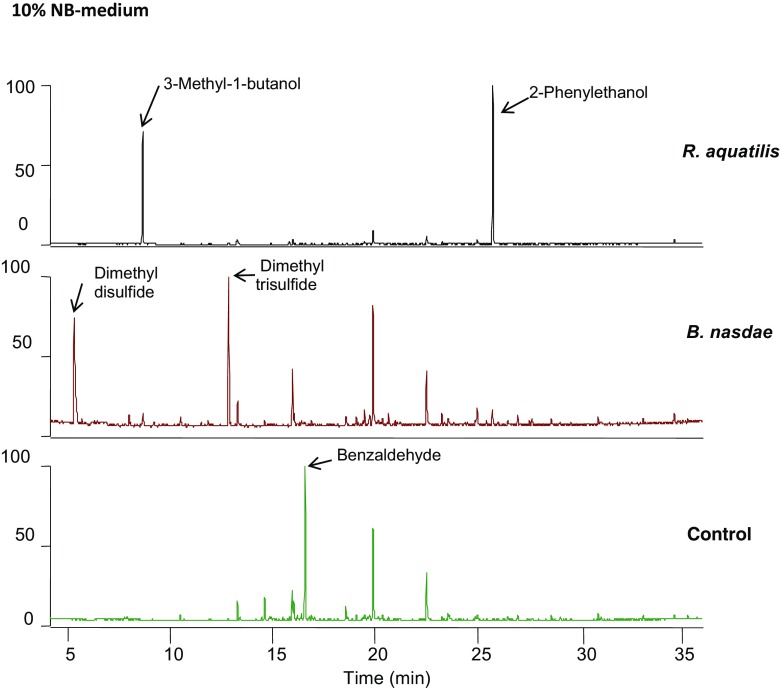
Volatile compounds detected by SPME-GC-MS in the headspace above cultures of bacteria isolates: *R. aquatilis* isolate Ha1 and *B. nasdae* isolate Ha2 grown in 10% NB medium. The figures show a large difference in compounds emitted from the two isolates, where *R. aquatilis* produces mainly 3-methyl-1-butanol and 2-phenylethanol and *B. nasdae* produces mainly dimethyl disulfide and dimethyl trisulphide. Benzaldehyde, detected above the sterile medium utilized as control, was not detected above either of the bacteria cultures

**Fig. 2:**
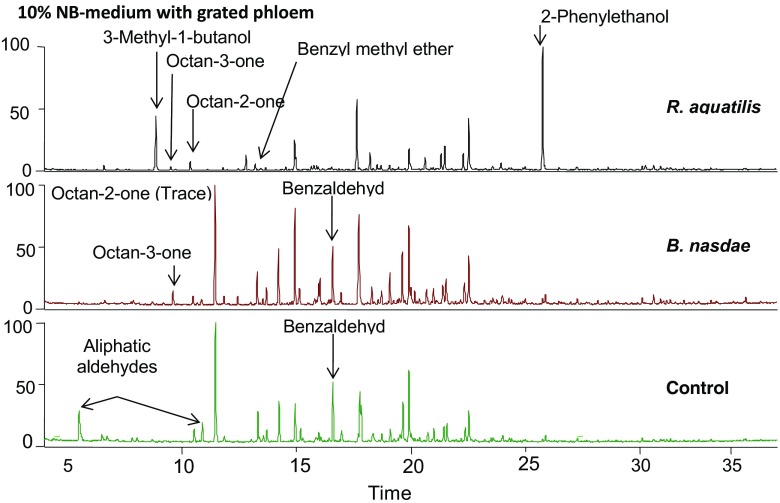
Volatile compounds detected by SPME-GC-MS in the headspace above cultures of bacteria isolates: *R. aquatilis* isolate Ha1 and *B. nasdae* isolate Ha2 grown in diluted NB-medium with grated phloem added. The chromatograms show a large difference in compounds detected from the two cultures, where *R. aquatilis* produces 3-methyl-1-butanol and 2-phenylethanol while the emission from the culture with *B. nasdae* is similar to that from the control except for the absence of aliphatic aldehydes

**Fig. 3:**
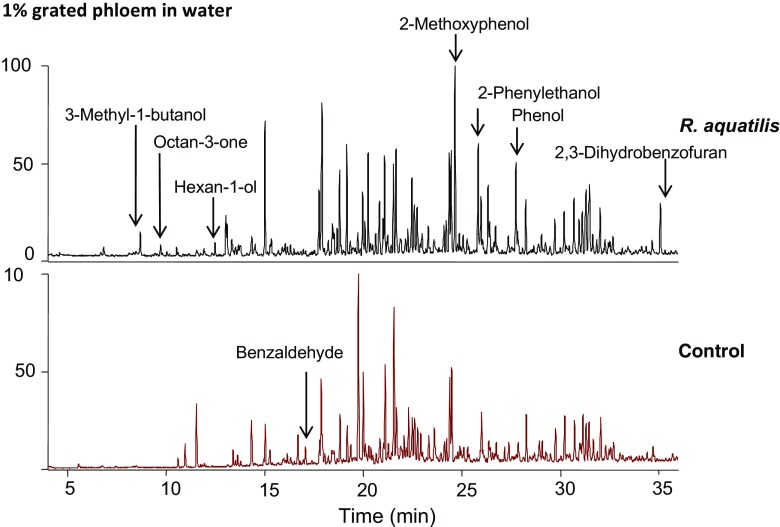
Representative chromatograms obtained by SPME-GC-MS showing the volatiles emitted from a sample with 1% grated phloem in water and the bacterium *R. aquatilis* isolate Ha3 added and the control sample with grated phloem in water only. Main compounds emitted from the sample are 3-methyl-1-butanol, 2-methoxyphenol, 2-phenylethanol, and 2,3-dihydrobenzofuran, and the main compound in the control is longipinene

After the first screening, the isolates of *R. aquatilis* and *B. nasdae* were grown in four types of media with different nutrient availabilities. After limiting the nutrient availability by growing the bacteria in (i) 10% diluted NB medium, *R. aquatilis* isolate Ha1 released two main compounds: 3-methyl-1-butanol and 2-phenylethanol (Fig. 1). In (ii) 10% diluted NB medium supplemented with grated bark, a possible natural source of nutrient for bacteria associated with a wood-feeding insect, *R. aquatilis* continued to produce 3-methyl-1-butanol and 2-phenylethanol as its main compounds, in addition to 2-octanone and 3-octanone (Fig. 2). When *R. aquatilis* was cultured on (iii) 1% grated bark in water, where bark constitutes the only nutrient source, the volatiles released from the mixture were oxygenated terpenoids, such as 3-methyl-1-butanol, 1-hexanol, and 3-octanone, structurally related aliphatic compounds, and aromatics such as 2-methoxyphenol (main compound), 2-phenylethanol, 2-phenylacetaldehyde, phenylmethanol, benzyl methyl ether, phenol, and 2,3-dihydrobenzofuran (Fig. 3). To confirm the production of aromatic compounds from the bacteria, ^13^C-6 ring-labeled phenylalanine was supplemented to diluted (iv) NB medium. In *R. aquatilis* isolate Ha3, five substances were found to be incorporated with the fully isotope-labeled benzene ring. The main compound, 2-phenylethanol, had a relative abundance of 96%, and 95% of its main fragments was incorporated with the isotope-labeled ring. The main fragment in the mass spectrum (m/z = 91) of 2-phenylacetaldehyde was 99% (m/z = 97) incorporated, phenylmethanol (M+ = 108) was 80% incorporated, benzaldehyde (M+ = 106) was 75% incorporated (112), and benzyl methyl ether (91) was 40% incorporated (Table 2).

**Table 2:** Labeled compounds emitted by *R. aquatilis* and *B. nasdae* when cultured on NB medium supplemented with ^13^C–ring-labeled L-phenylalanine

Compounds	*R. aquatilis* Proportion of labeled compounds (% incorporation)	*B. nasdae* Proportion of labeled compounds (% incorporation)
Benzyl methyl ether	0.7 (40)	
Benzaldehyde	0.1 (75)	30 (75)
Phenylacetaldehyde	1.4 (99)	
Phenylmethanol	1.8 (80)	45 (40)
2-Phenylethanol	96 (95)	25 (55)

In (i) 10% diluted NB medium, *B. nasdae* released mainly oligosulfides as dimethyl disulfide and dimethyl trisulfide, an alkylpyrazine, as well as minor amounts of phenylmethanol and 2-phenylethanol (Fig. 1). In (ii) 10% diluted NB medium supplemented with grated bark, the emission from the *B. nasdae* culture was similar to that from the control except for the absence of the aliphatic aldehydes with retention times 5.49 and 10.87 (Fig. 2). When *B. nasdae* was cultured on (iii) 1% grated phloem in water, only trace amounts of aromatics were found and the chemical profile of the phloem-water mixture closely resembled the control comprising mainly terpenoids (Fig. 3). Only traces of dimethyloligosulfides were detected.

In the stable isotopic labeling experiment, *B. nasdae* emitted only minor amounts of volatiles, which consisted mainly of benzaldehyde and phenylmethanol. Furthermore, the incorporation of the aromatic ^13^C-labeled ring structure in 2-phenylethanol was low in *B. nasdae* compared to *R. aquatilis* isolate Ha3 (Table 2).

### Antifeedant Test

**Fig. 4:**
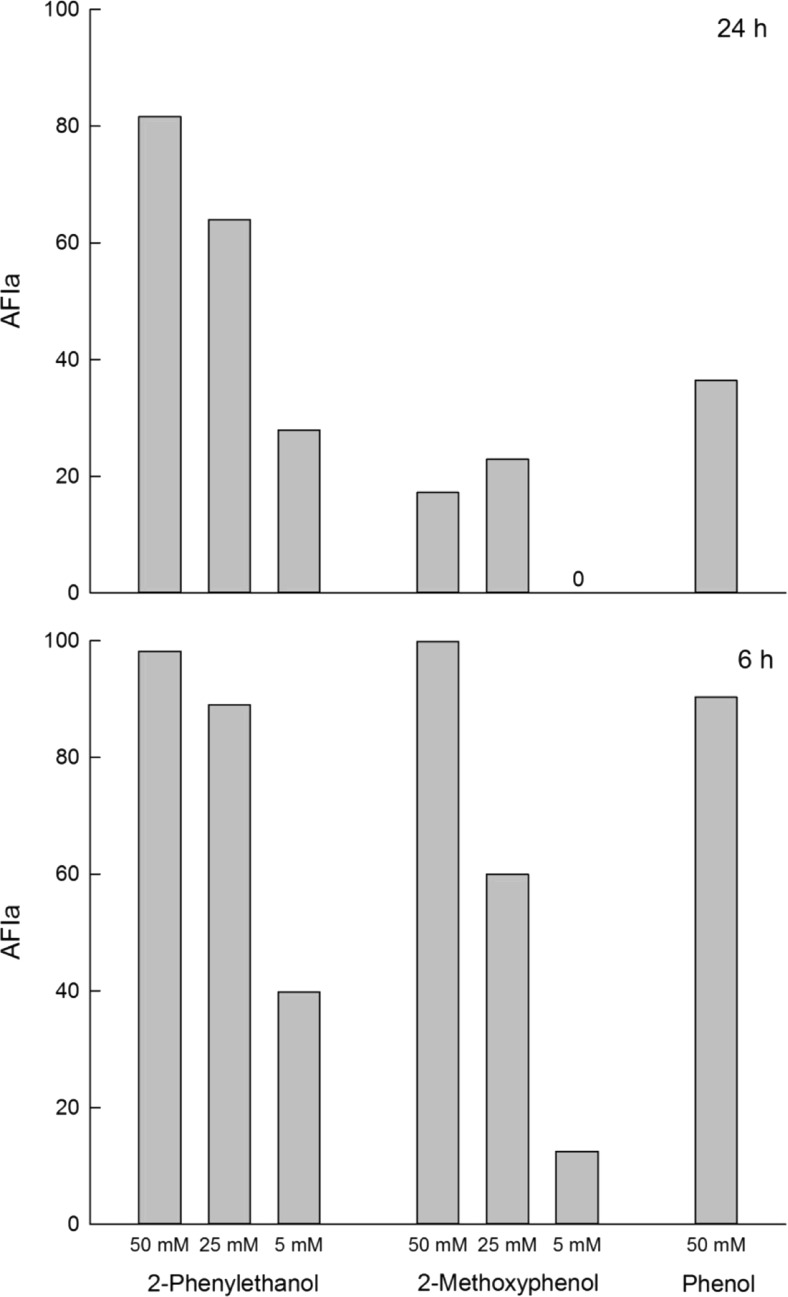
An antifeedant effect is seen after 6 h and 24 h on adult pine weevils (*n* = 40; 20 females and 20 males) by three compounds identified in the emissions of *R. aquatilis*. Positive values of AFIa (up to a maximum of 100) reflect an antifeedant effect

The main compounds produced of the isolated bacteria strains, 2-phenylethanol and 2-methoxyphenol, showed strong antifeedant effect on the pine weevils that lasted for the entire 24-h test period (Fig. 4). A clear dose-response effect of AFIa was found for the three concentrations tested (50, 25, and 5 mM) after 24 h. Phenol, also present in the majority of isolates isolated, was tested at a 50 mM concentration and showed a shorter-lasting antifeedant effect with a very high AFIa value (90) after 6 h that decreased to just 36 after 24 h. Weevils of both sexes responded similarly to the compounds tested.

## Discussion

Pine weevil females cover their deposited eggs with feces, which have been proposed to protect the egg from being eaten by conspecifics [10]. The aim of this study was to find the source for the antifeedant compounds present in pine weevil feces. Since bacteria that could produce antifeedants were a possible source, we isolated bacteria from pine weevil guts and identified them based on their 16S rRNA gene sequences. Our results show that the culturable bacteria belonged to a distinct community with a high proportion of *R. aquatilis.* Other bacterial species such as *B. nasdae*, *K. kristinae*, and *M. terreus* were also identified. Bacteria within the genus *Rahnella* are known as endophytes on spruce [28]*. R. aquatilis* is also identified as a dominating bacterium together with *Serratia liquifaciens* and *Serratia plymuthica* in larvae and adults of the mountain pine beetle *Dendroctonus valens* [29].

A large diversity of chemical compounds are known to be produced by bacteria, ranging from highly volatile aliphatic alcohols, pyrazines, and sulfides to less volatile aromatic alcohols and phenolics [30–32]. The emissions of volatiles from the bacteria in this study were highly dependent on the substrate. For example, alkylpyrazines and dimethyloligosulfides were present in the volatiles emitted from the bacteria when growing on nutrient-rich medium. Pyrazines have earlier been identified from a *Paenibacillus polymyxa* isolate [33] and from *Chondromyces crocatus* [30]. The dimethyloligosulfides have been identified in the volatiles emitted by both Gram-positive and Gram-negative bacteria [30, 34–41].

The aliphatic component 3-octanone, present in a typical mushroom scent, was emitted from the *R. aquatilis* isolates, which also emitted 2-octanone and 1-nonanol, the latter two showing antifeedant properties for pine weevils (M. Azeem, personal communication).

The major compounds released from *R. aquatilis* when grown in rich media (10% NB) were 2-phenylethanol and 3-methyl-1-butanol. 3-Methyl-1-butanol, 1-nonanol, and the aromatic alcohol 2-phenylethanol have previously all been found in the emissions of bacteria [34, 37–40, 42–44]. These compounds are also released by *Pantoea* strains isolated from the gut of *Anopheles gambiae* grown in saline media (0.7% NaCl in autoclaved water) [31]. Notably, 2-phenylethanol is released from the bark of *P. sylvestris* during pine weevil feeding (Lundborg et al. 2016).

When adding the isolated bacteria *R. aquatilis* and *B. nasdae* to the bark medium in water without any further nutrition, the volatile profile of the water-phloem mixture mainly changed for *R. aquatilis* isolate Ha3. From constituting mainly oxygenated mono-, sesqui-, and diterpenes, a number of aromatic compounds appeared in the headspace. *R. aquatilis* is thus an interesting isolate for further investigations as it produces a large number of aromatic compounds with potential antifeedant properties.

By culturing the two species *R. aquatilis* and *B. nasdae* producing the most different volatile profiles in this study when growing on grated phloem, we could show different utilizations of L-phenylalanine. By adding ^13^C–ring-labeled amino acid to the medium, we could verify that *Rahnella* bacteria more efficiently utilized phenylalanine as a precursor for producing a number of putative antifeedants. Thus, this bacterium might be effective in producing antifeedants from the free amino acid present in the bark phloem. In addition, we compared the proportion and incorporation of stable isotopes of the bacteria-biosynthesized aromatic compounds. However, the major compound, 2-methoxyphenol, was not labeled in the isotope labeling experiments. Thus, it may originate from either constitutive polyphenols or lignin present in the grated phloem.

One of the major compounds produced by bacteria in the grated phloem-water solution was 2-phenylethanol which was also shown to be a strong antifeedant for the pine weevils. This compound was also identified in volatiles from pine weevil feces (M. Azeem, personal communication). 2-Methoxyphenol and phenol also elicited strong but shorter-lasting effects. Aromatic compounds previously identified in the feces, like 4-methylphenol [10] and 1-methoxy-4-methylbenzene, have shown a short-time antifeedant effect when applied on test twigs [10]. The short-lasting effects of these molecules are most probably due to their higher volatility compared to 2-phenylethanol. 1,4-Dihydroxybenzene which is present in the feces was a stronger antifeedant (AFIa = 0.72) than the corresponding methoxy derivative, 1,4-dimethoxybenzene (AFIa = 0.45) after 24 h feeding [10]. Aromatic compounds like ethyl, isopropyl, propyl, and butyl esters of cinnamic acid have earlier shown to be efficient antifeedants [10, 12–15]. Of these, the most effective substance chemically related to constituents in the feces is the ethyl ester (AFIa = 0.83 after 24 h) and isopropyl ester of cinnamic acid (AFIa = 0.96 after 24 h), which have strong antifeedant properties and a long-lasting effect [12].

The microorganisms in the gut and their volatiles released during microbial degradation of the phloem are chemically highly diverse and might influence the behavior of the pine weevil. In the case presented here, there are possible effects on the survival of eggs and larvae. The molecules showing a short-lasting effect in the antifeedant test are continuously produced by microbes, and therefore, the mixture of nibbled phloem and feces produced by the female pine weevil to cover the egg will sustain an antifeedant effect as long as the microbial degradation of the phloem is going on, despite the short-lasting effect. In this study, we focused on analyzing the volatiles emitted during temperatures with the optimal growth phase anticipating a high activity of the bacteria. While these temperatures are recorded during summer time in the clear-cuts where pine weevils lay their eggs, it is possible that other temperatures may yield a different set of volatile compounds emitted as bacteria react to different temperatures with different metabolic pathways [45]. Pine weevils feed at temperatures between 5 and 30 °C with the physiological optimum at 25 °C [46]. The feeding activity of pine weevils is also regulated by the photoperiod with most of the feeding taking place during night and morning [47]. Probably most of the feeding occurs when the weevils experience temperatures in the range of 15–22 °C. A detailed investigation of the thermo-regulated switches in bacteria isolated from pine weevils potentially affecting volatile emissions may be a future direction of our studies.

In this study, we investigated the culturable pine weevil gut microbial community and identified a possible microbial source for the pine weevil antifeedants that may protect the egg during the entire period until it hatches. This study increased our understanding of how metabolites from microbes associated with the pine weevil may have an influence on the behavior of the pine weevils. Only microbes that could utilize the natural source, pine phloem, and start to produce antifeedants or deterrent compounds within a few hours were of further interest. Our results also show that *R. aquatilis* might be a useful microorganism for future pilot studies of the bioproduction of pine weevil antifeedant blend, to be used for the protection of conifer seedling when planted in the field.
